# Structural Characterization of Unusual Fatty Acid Methyl Esters with Double and Triple Bonds Using HPLC/APCI-MS^2^ with Acetonitrile In-Source Derivatization

**DOI:** 10.3390/molecules26216468

**Published:** 2021-10-26

**Authors:** Petra Horká, Vladimír Vrkoslav, Jiří Kindl, Karolina Schwarzová-Pecková, Josef Cvačka

**Affiliations:** 1 Institute of Organic Chemistry and Biochemistry of the Czech Academy of Sciences, Flemingovo Náměstí 542/2, 166 00 Prague 6, Czech Republic; peta.machajda@gmail.com (P.H.); vladimir.vrkoslav@uochb.cas.cz (V.V.); kindlj@ftz.czu.cz (J.K.); 2 Department of Analytical Chemistry, Faculty of Science, Charles University, Hlavova 2030/8, 128 43 Prague 2, Czech Republic; karolina.schwarzova@natur.cuni.cz

**Keywords:** acetonitrile-related adducts, acetylenic lipids, double and triple bond localization, in-source derivatization, mass spectrometry

## Abstract

Double and triple bonds have significant effects on the biological activities of lipids. Determining multiple bond positions in their molecules by mass spectrometry usually requires chemical derivatization. This work presents an HPLC/MS method for pinpointing the double and triple bonds in fatty acids. Fatty acid methyl esters were separated by reversed-phase HPLC with an acetonitrile mobile phase. In the APCI source, acetonitrile formed reactive species, which added to double and triple bonds to form [M + C_3_H_5_N]^+•^ ions. Their collisional activation in an ion trap provided fragments helpful in localizing the multiple bond positions. This approach was applied to fatty acids with isolated, cumulated, and conjugated double bonds and triple bonds. The fatty acids were isolated from the fat body of early-nesting bumblebee *Bombus pratorum* and seeds or seed oils of *Punicum granatum*, *Marrubium vulgare*, and *Santalum album*. Using the method, the presence of the known fatty acids was confirmed, and new ones were discovered.

## 1. Introduction

The localization of double and triple bonds (DBs and TBs) is a key step in the structural characterization of fatty acids (FAs). The biological functions of lipids are often linked to the specific arrangement of multiple bonds in their FA chains. Lipids with unusually arranged double bonds and triple bonds are of interest because of their function in living organisms and their unique biological effects and potential use in medicine [1,2]. Mass spectrometry is useful for pinpointing the double bonds in FAs and their methyl esters (FAMEs), especially when combined with chromatography. The early methods were based on the electron ionization of derivatized lipids. Derivatization, either at the double bond site or at the carboxylic group, is required because of the bond migration along the aliphatic chains during electron ionization. Numerous FA derivatives, including pyrrolidides [3], 4,4-dimethyloxazoline (DMOX) [4], or dimethyl disulfide (DMDS) derivatives [5], have found their use in GC/MS. Later, HPLC/MS-based methods began to be developed. Unlike GC/MS, these methods also make it possible to analyze less volatile and non-volatile FAs and their derivatives. A number of methods have been proposed for localizing double bonds using electrospray ionization, including Paternò–Büchi photochemical derivatizations [6,7,8], epoxidation in low-temperature plasma [9,10] and negative-ion paper-spray ionization [11], post-column epoxidation and peroxidation [12], charge-switch derivatization with *N*-(4-aminomethylphenyl) pyridinium (AMPP) [13,14], or ozone-induced dissociation (OzID) [15,16], and combining charge-switch derivatization with OzID [17].

Besides electrospray ionization, atmospheric-pressure chemical ionization (APCI) can also be applied for localizing double bonds in HPLC/MS [18,19,20,21,22,23]. The methods rely on acetonitrile-related reactive species formed in the ion sources. The use of even-electron (1-methyleneimino)-1-ethenylium as a reagent for derivatizing double bonds was initially developed for chemical ionization [24,25,26,27] and later applied in APCI-MS [18]. Using helium as a nebulizing gas, C_3_H_4_N^+^ adducts ([M + 54]^+^) of triacylglycerols (TGs) were formed, and their CID spectra indicated the positions of the original double bonds [18]. Later, we showed that APCI sources operated under conventional conditions with nitrogen nebulizing gas yield odd-electron C_3_H_5_N^+•^ adducts ([M + 55]^+•^) [19]. The collision activation of the adducts induced cleavages of C–C bonds next to the original double bond, leading to pairs of diagnostic fragments indicating the double bond position. The advantage of this approach lies in its simplicity: the only requirement for an HPLC/APCI-MS^2^ method is the presence of acetonitrile in the mobile phase. The method has been applied for the structure elucidation of various unsaturated lipids, including FAMEs [20,28], hydroxy-FAMEs [23], wax esters [19], diol diesters [22], or TGs [21].

To date, only a few methods for determining the position of triple bonds in lipids have been published [27,29,30,31,32]. Triple bonds in FAs can be pinpointed after DMOX derivatization using GC/MS [31]. While a conjugated system of double bonds manifests itself by a series of fragments differing by 12 Da, triple bond-related fragments differ by 10 Da. It allows for the structural characterization of conjugated ene–yne acids. Still, the fragmentation of conjugated yne–yne or yne–yne–ene bonds is more complex, and the spectra are difficult to interpret [30]. Using this approach, many acetylenic lipids have been identified in plants [29,30,32]. The position of a triple bond can also be determined using acetonitrile chemical ionization based on (1-methyleneimino)-1-ethenylium adducts formation [27]. To the best of our knowledge, no method for localizing triple bonds using HPLC/MS has appeared in the literature so far.

Double bond positions in FAs reflect specificities of desaturases involved in their biosynthesis. Most monounsaturated FAs have a double bond in 9-position. Other positions are also relatively common, for instance, 7-position in algae, 5- and 10-positions in bacteria, or 6-position in plants [33]. Double bonds in polyunsaturated FAs are typically spaced by one methylene group (methylene interrupted). FAs with double bonds separated by two or more methylene units are found, for instance, in marine sponges *Microciona prolifera* (FA 26:2n-17,21 and FA 26:3n-7,17,21) [34,35], *Dysidea fragilis* (FA 25:3n-8,16,20; FA 25:3n-6,16,20; FA 24:3n-7,15,19 and FA 24:2n-7,17) [36], or *Hymeniacidon sanguinea* (e.g., FA 28:2n-9,19,23; FA 26:2n-17,21; FA 26:3n-7,17,21; FA 24:2n-15,19 and FA 24:3n-7,15,19) [37]. More than twenty different FAs with double bonds separated by two or more methylene units were identified in the gonads of limpets *Cellana grata* [38], *Collisella dorsuosa* [38], and *Cellana toreum* [39,40]. Unusual FAs with 24, 26, and 28 carbon atoms were found in TGs isolated from the fat body of early-nesting bumblebee *Bombus pratorum*. FA 26:2n-7*c*,17*c* occupied one, two, or all three positions in the TGs [41].

FAs with a conjugated system of double bonds are mostly represented by conjugated linoleic acids (CLAs) and conjugated linolenic acids (CLnAs), which are collective terms for the positional and geometric isomers of octadecadienoic and octadecatrienoic acids, respectively. CLAs exist naturally at higher concentrations in animal products, such as milk fat, cheese, and ruminant meat [42,43]. Two double bonds in CLAs are primarily in positions 9 and 11, or 10 and 12, and each of the double bonds can be either *cis* or *trans* [44]. CLAs are important for human nutrition. For instance, *cis*-9,*trans*-11 and *trans*-10,*cis*-12 isomers reduce carcinogenesis and atherosclerosis, increase bone and muscle mass, and exhibit antidiabetic effects [42,45]. CLnAs are found in plant seed oils, including oils from *Vernicia fordii* (α and β-eleostearic acid; FA 18:3n-5*t*,7*t*,9*c* and FA 18:3n-5*t*,7*t*,9*t*) [46], *Catalpa bignonoides* (catalpic acid; FA 18:3n-5*c*,7*t*,9*t*) [47], *Jacaranda mimosifolia* (jacaric acid; FA 18:3n-6*c*,8*t*,10*c*) [48], or *Calendula officinalis* (alfa-calendic acid; FA 18:3n-6*c*,8*t*,10*t* and beta-calendic acid; FA 18:3n-6*t*,8*t*,10*t* [49]. A rich source of CLnA is pomegranate (*Punicum granatum*) seed oil (PSO). It contains punicic acid (FA 18:3n-5*c*,7*t*,9*c*), α-eleostearic acid (18:3n-5*t*,7*t*,9*c*), β-eleostearic acid (18:3n-5*t*,7*t*,9*t*), and catalpic acid (18:3n-5*c*,7*t*,9*t*) [50,51,52,53,54]. The structures of several other CLnAs in PSO remain to be clarified [51,55]. CLnAs are known for their antioxidant, anti-inflammatory, anti-atherosclerotic, antitumor, and serum lipid-lowering activities. They help fight against cancers, obesity, diabetes, and heart diseases [53,56,57].

Lipids with two cumulated double bonds (allenic lipids) are found in Lamiaceae family plants; elsewhere in nature, they are rare [58,59]. The first known C18 allenic FA, laballenic acid (FA 18:2n-12,13), was isolated from *Leonotis nepetaefolia* seed oil [60] and later reported also from other Lamiaceae species [61]. Lamenallenic acid (FA 18:3n-2*t*,12,13) was discovered in *Lamium purpureum* seed oil [62]. Phlomic acid (FA 20:2n-12,13) was found in several species of *Phlomis* genus (Lamiaceae) [61]. Seeds of *Marrubium vulgare* contain laballenic acid and phlomic acid [59]. Allenic lipids are known for their anticancer, anti-inflammatory, antiviral, and antibacterial activities [1].

FAs with triple bonds (acetylenic FAs) are relatively widely distributed in nature. They are found in plants, fungi, microorganisms, and invertebrates [58,63,64,65]. FAs and other acetylenic lipids in plants serve as chemical protection against microorganisms. They are toxic to bacteria, viruses, and insects [2,66,67,68,69]. Many acetylenic lipids exhibit fungicidal, phototoxic, antitumor, and other properties [1], which render them potentially useful in medicine. The chain length and triple bond positions affect their fungicidal properties [2,68]. The structures and cytotoxic activities of acetylenic lipids were reviewed recently [70]. Some plant FAs contain triple and double bonds conjugated, e.g., pyrulic acid (FA 17:2n-7,9^TB^), ximenynic (also termed santalbic) acid (FA 18:2n-7*t*,9^TB^), or heisteric acid (FA 18:3n-7*t*,9^TB^,11*c*) from *Heisteria silvanii* seed oil [32]. *Ximenia americana* contains FA 18:1n-13^TB^ and FA 18:4n-2,4,8,6^TB^ [2,66]. Santalbic acid (FA 18:2n-7*t*,9^TB^), identified for the first time in *Santalum album* [58,63], is one of the few acetylenic FAs occurring at higher levels in plants. It is found in the seed oils of the Santalaceae, Olacaceae, and Opiliaceae families, where it can reach up to 95% of the total FAs [71,72]. Other biologically active acetylenic acids are crepenynic acid (FA 18:2n-6^TB^,9*c*), tariric acid (FA 18:1n-12^TB^), stearolic acid (FA 18:1n-9^TB^), or nonadec-6-ynoic acid (FA 19:1n-12 ^TB^) [67,73,74,75]. FAs with a triple bond can also be found in water mosses [40,76,77,78,79].

This work deals with the localization of double and triple bonds in FAMEs. The conversion of lipids or lipid mixtures to FAMEs is frequently used in lipidomics workflows because the GC or LC analysis of FAMEs provides quick and valuable information on the fatty acyl chains. Here, FAME standards and FAMEs obtained by the transesterification of the TGs from biological samples were analyzed by HPLC/APCI-MS/MS using an acetonitrile mobile phase. Isolated, cumulated, and conjugated double bonds and triple bonds were localized using the fragmentation of [M + C_3_H_5_N]^+^^•^ adducts generated in the ion source. To the best of our knowledge, the localization of triple bonds in FAMEs by RP-HPLC with MS detection is reported here for the first time.

## 2. Results and Discussion

The chromatographic separation of FAMEs was achieved on the Develosil RP-Aqueous C30 column using isocratic elution with acetonitrile. The mobile phase in the APCI source formed reactive species, which added to double and triple bonds. The adducts were isolated and activated in the ion trap to generate ions bearing information on the original double or triple bond position. The diagnostic ions formed by the cleavages of adjacent C–C bonds were marked α if they carried the ester moiety or ω if they contained the terminal-carbon end without the ester group. The diagnostic peaks corresponding to cleavages before the first and after the last unsaturated bond in polyunsaturated FAMEs tended to be more abundant than the others. This phenomenon was used for deducing the arrangement of the double and triple bonds in polyunsaturated chains. A parameter named “multiple bond region” (MBR) was calculated and tabulated for various theoretically possible arrangements of multiple bonds (Table 1). The MBR value was calculated using theoretical *m/z* values of the adduct (precursor) and α and ω fragments corresponding to cleavages before the first and after the last unsaturated bond as follows:

(1) $$\mathrm{MBR}=m/z\;\left(\alpha \right)+m/z\;\left(\omega \right)-m/z\;\left({\left[M+55\right]}^{+•}\right)$$

The experimental MBR values calculated for the adduct and the most abundant α and ω fragments in the spectra were then compared to theoretical MBRs. For instance, the MS/MS spectrum of [M + 55]^+^^•^ adduct of unknown FA at *m/z* 347.0 provided the most abundant α and ω peaks at *m/z* 290.2 and *m/z* 190.2, respectively. The calculated MBR value (290 + 190 − 347 = 133) suggested FAME with three conjugated double bonds (Table 1). Diagnostic ions were accompanied by less abundant satellite peaks differing from α and ω ions by 14 or 15 Da. These fragments representing cleavages at more distant C–C bonds were important for distinguishing double and triple bonds. The elemental composition of the major fragments in the spectra of FAME standards was confirmed by Orbitrap high-resolution data (Supplementary Materials Table S1).

### 2.1. Mass Spectra of Standards with Conjugated Double Bonds

**Figure 1:**
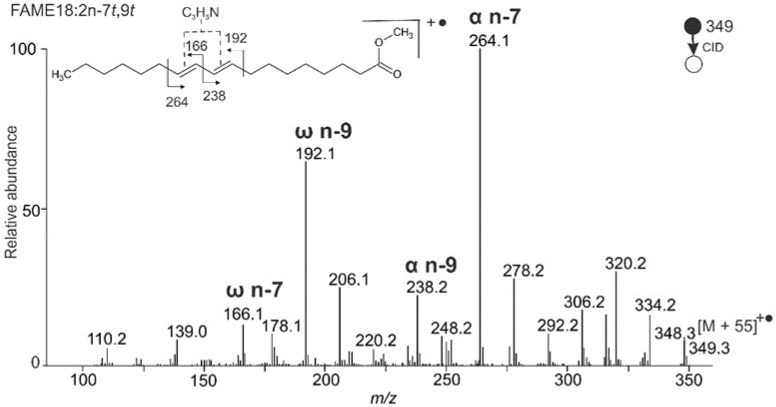
APCI MS/MS CID spectrum of [M + 55]^+^^•^ adduct of Mangold’s acid methyl ester (FAME 18:2n-7*t*,9*t*); MBR = 264 + 192 − 349 = 107.

The system with two conjugated double bonds was investigated using standards of FAME 18:2n-7*t*,9*t* (Mangold’s acid methyl ester) and FAME 18:2n-7*c*,9*c* (ricinenic acid methyl ester). The fragments in the MS/MS spectrum for FAME 18:2n-7*t*,9*t* (Figure 1) were rationalized as follows: α n-7 peak at *m/z* 264.1, α n-9 peak at *m/z* 238.2, ω n-7 peak at *m/z* 166.1, and ω n-9 peak at *m/z* 192.1. The MBR value calculated from the two most intense fragments in the spectrum (i.e., *m/z* 192.1 and *m/z* 264.1) was 107. Despite the presence of satellite fragments differing by 14 Da from the diagnostics peaks, the spectrum provided clear evidence of two conjugated double bonds in the n-7 and n-9 positions. The spectrum of FAME 18:2n-7*c*,9*c* having the opposite geometry on both double bonds looked similar (Figure S1), which confirmed the negligible effect of double bond geometry on the adduct fragmentation documented earlier [19].

**Figure 2:**
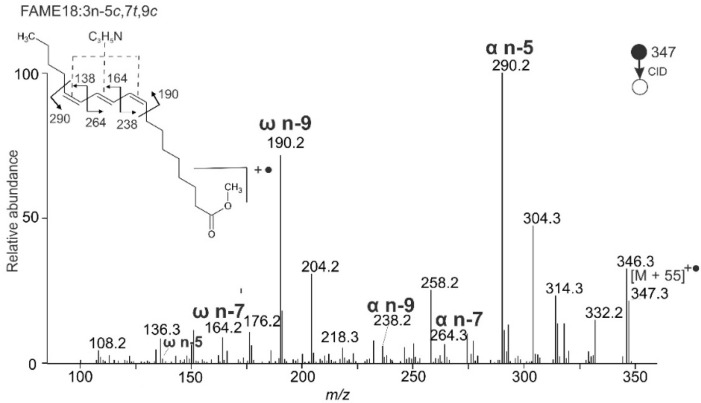
APCI MS/MS CID spectrum of [M + 55]^+^^•^ adduct of punicic acid methyl ester (FAME 18:3n-5*c*,7*t*,9*c*); MBR = 290 + 190 − 347 = 133.

The MS/MS spectrum of punicic acid methyl ester with three conjugated double bonds (FAME 18:3n-5*c*,7*t*,9*c*) is shown in Figure 2. The major fragments in the spectrum were formed by cleavages before and after the series of double bonds. They were easily distinguishable from the other ions. The most abundant fragments α n-5 at *m/z* 290.2 and ω n-9 at *m/z* 190.2 delimited the group of conjugated double bonds and corresponded to an MBR value of 133. The fragments formed by the cleavages between conjugated double bonds α n-7 (*m/z* 264.3), α n-9 (*m/z* 238.2), ω n-7 (*m/z* 164.2), and ω n-5 (*m/z* 138.2) were of low intensities but discernable in the spectrum. The same diagnostic fragments and MBR value could theoretically be expected for a FAME with two cumulated double bonds separated by one methylene group from the third double bond. Such an arrangement of double bonds would be, however, clearly distinguishable because the system of cumulated double bonds manifests itself by abundant α + 1 Da ion (Section 2.3.3). Such an ion (*m/z* 251 or *m/z* 291 in this case) is not present in the spectrum. Therefore, the spectrum in Figure 2 can be unambiguously interpreted as FAME 18:3n-5,7,9.

### 2.2. Mass Spectra of Standards with a Triple Bond

**Figure 3:**
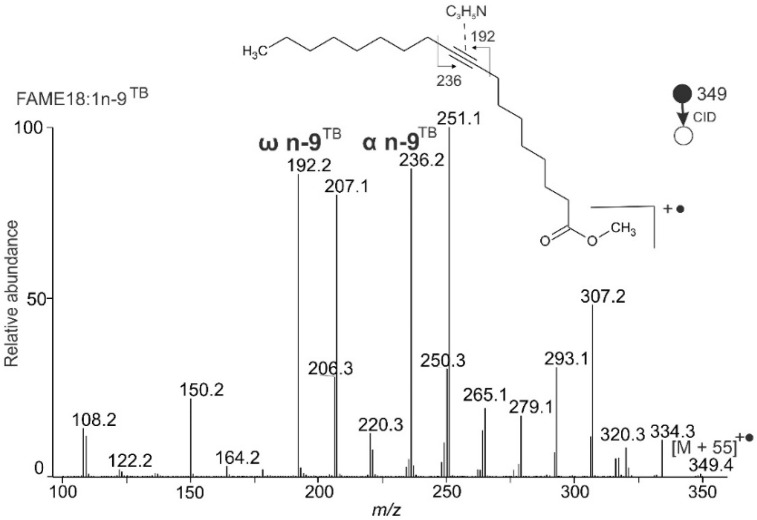
APCI MS/MS CID spectrum of [M + 55]^+^^•^ adduct of stearolic acid methyl ester (FAME 18:1n-9^TB^); MBR = 236 + 192 − 349 = 79.

Figure 3 shows the MS/MS spectrum of FAME 18:1n-9^TB^ (stearolic acid methyl ester) [M + 55]^+^^•^ adduct. The abundant fragments *m/z* 236.2 (α n-9^TB^) and *m/z* 192.2 (ω n-9^TB^) clearly indicated a triple bond in the n-9 position. Unlike FAMEs with double bonds, the satellite fragments differed by +15 Da from α^TB^ and ω^TB^ (*m/z* 207.1 and *m/z* 251.1, respectively). The intensities of the diagnostic fragments and their +15 Da satellites were similar, allowing us to recognize these peaks in the spectrum easily. Such a pattern distinctly indicated a triple bond. Satellite fragments differing by +14 Da, typical for double bonds, were present at significantly lower intensities.

**Figure 4:**
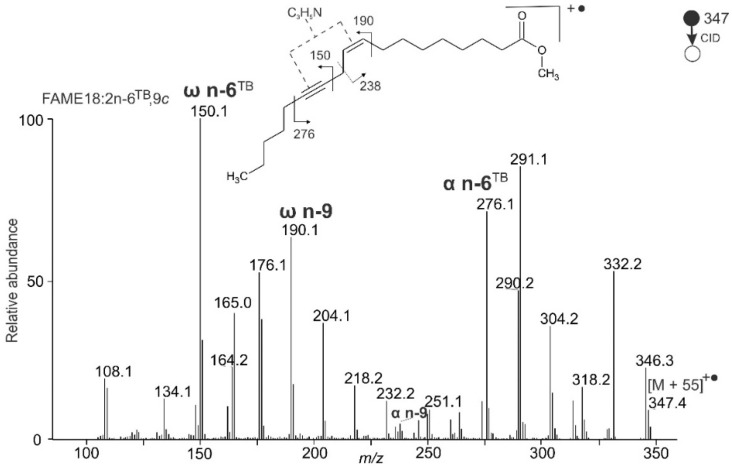
APCI MS/MS CID spectrum of the [M + 55]^+^^•^ adduct of crepenynic acid methyl ester (FAME 18:2n-6^TB^,9c); MBR = 276 + 190 − 347 = 119.

The satellite fragment ions made it also possible to characterize FAMEs with a combination of double and triple bonds. For instance, crepenynic acid methyl ester with one double bond and one triple bond (FAME 18:2n-6^TB^,9c) provided a spectrum with the most abundant peak at *m/z* 150.1 (Figure 4). This signal is a diagnostic fragment for triple bond (ω n-6^TB^) because its satellite appears at a 15 Da higher *m/z* value (*m/z* 165.0). Analogously, the *m/z* 276.1 with its satellite at *m/z* 291.1 is the triple bond diagnostic peak (α n-6^TB^). Fragment *m/z* 190.1 indicates a double bond (ω n-9) because its satellite peak appears at *m/z* 204.1.

### 2.3. Analysis of Natural Samples

The fragmentation of FAME standards with various arrangements of double and triple bonds helped us characterize the FAMEs isolated from biological samples. The identification procedure was initiated by deducing the number of carbons and level of unsaturation from the *m/z* values of the protonated FAMEs. The second step examined the MS/MS spectra of [M + 55]^+•^ ions to identify the diagnostic fragments and their satellites. The *m/z* values of the diagnostic fragments, MBR values, and the mass difference between the diagnostic fragments and satellites were used to deduce the positions of double and triple bonds. Finally, the retention times were checked for their consistency with the expected elution order of FAMEs [80,81].

#### 2.3.1. FAMEs from the Fat Body of *Bombus pratorum*

The early-nesting bumblebee *Bombus pratorum* is widespread in Europe. It is one of the earliest bumblebee species to emerge from hibernation each year. The fat body of *B. pratorum* males contains TGs with long, diunsaturated fatty acyls, which are structurally related to its marking pheromone [41].

**Figure 5:**
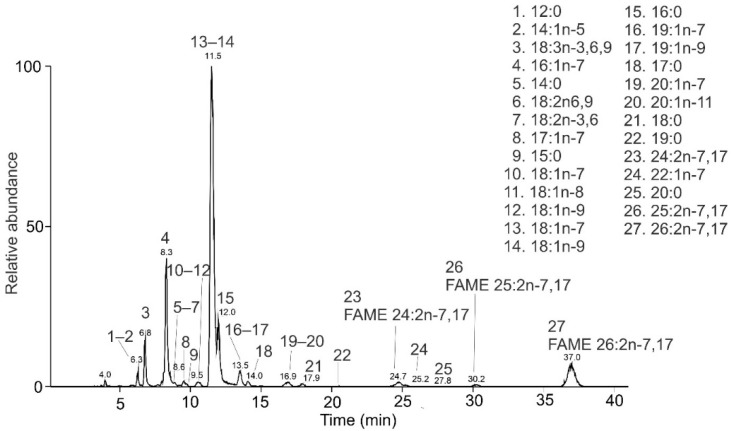
HPLC/APCI-MS base-peak chromatogram of FAMEs from the fat body of *Bombus pratorum* and the list of identified species.

**Figure 6:**
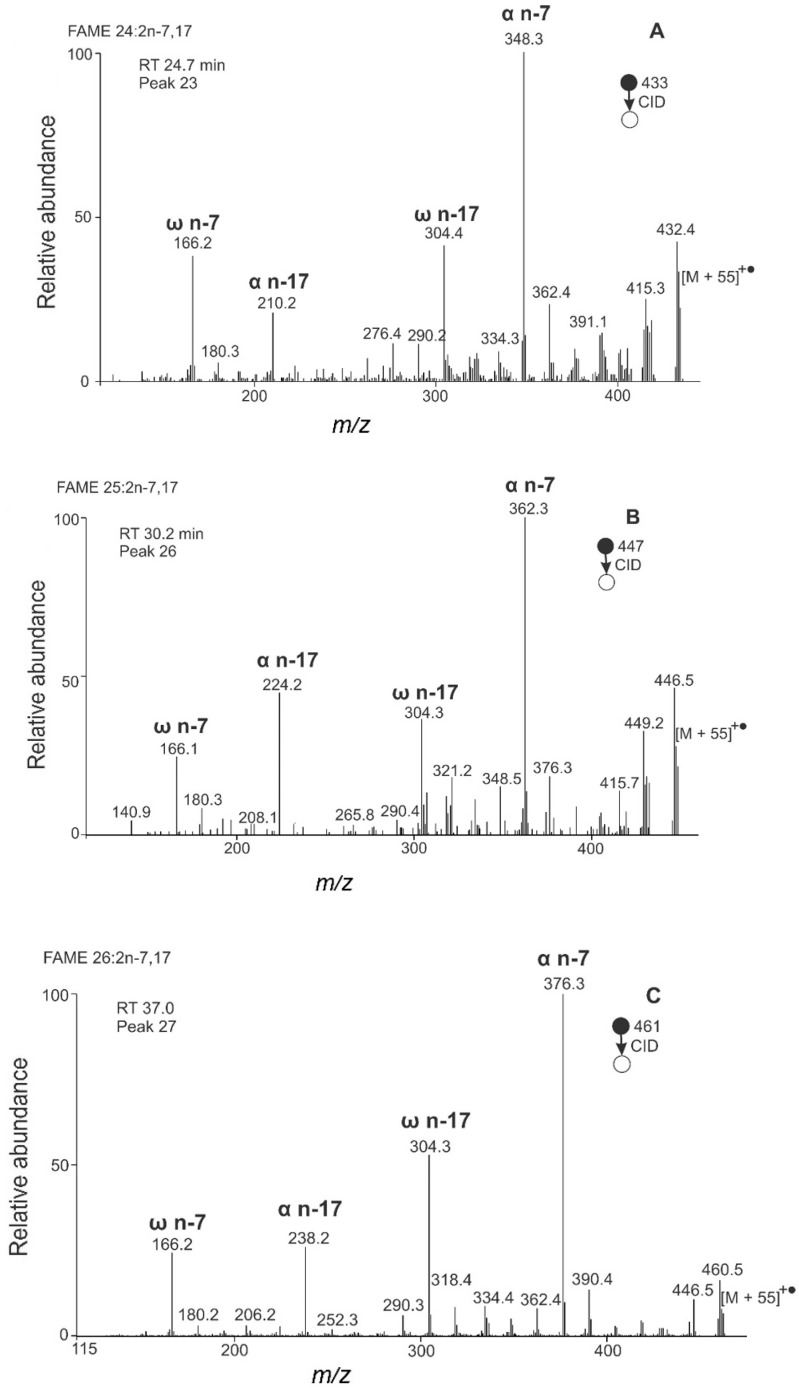
APCI MS/MS spectra of the [M + 55]^+^^•^ adducts of FAME from *B. pratorum* interpreted as FAME 24:2n-7,17 (**A**), 25:2 n-7,17 (**B**), and 26:2n-7,17 (**C**).

The chromatogram of *B. pratorum* FAMEs is shown in Figure 5. The MS/MS spectra of diunsaturated FAMEs (Figure 6) provided abundant and recognizable α and ω fragments interpreted as FAME 24:2n-7,17, FAME 25:2n-7,17, and 26:2n-7,17. The double bond positions were in excellent agreement with previous work, where the positions of the double bonds were established using dimethyl disulfide derivatization [41].

Altogether, nine saturated, fourteen monounsaturated, five diunsaturated, and one triunsaturated FAMEs were detected (Table 2). Nine of them (FAME 17:1n-7; FAME 17:0; FAME18:2n-3,6; FAME 18:1n-8; FAME 19:1n-7; FAME 19:0; FAME 22:1n-7; FAME 23:0; FAME 25:2n-7,17) are reported here for *B. pratorum* for the first time. To the best of our knowledge, FA 25:2n-7,17 has not been mentioned in the literature so far. FAMEs 25:2 are very rare in nature; the only known source of such acids are marine sponges producing different isomers [37,82,83,84]. FA 25:2n-7,17 likely serves as a precursor for tetracosadiene, a minor component (0.02 to 0.3%; I. Valterová 2021, personal communication, 18 April) of *B. pratorum* males’ secretion. Bumblebee males use the secretion to mark their patrolling routes [85].

#### 2.3.2. FAMEs from Pomegranate Seed Oil

**Figure 7:**
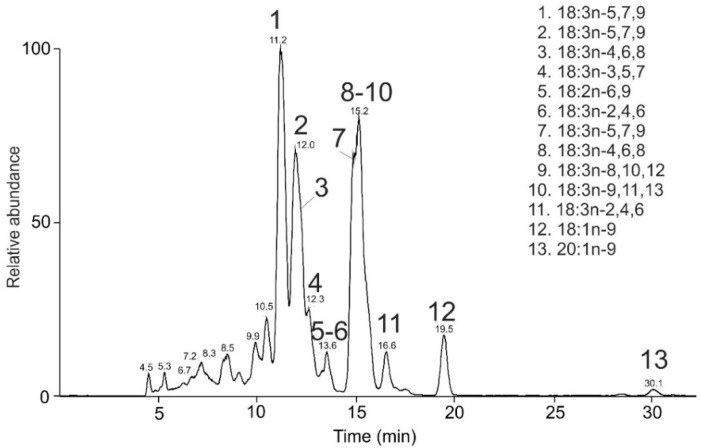
HPLC/APCI-MS base-peak chromatogram of FAMEs obtained from *Punicum granatum* seed oil and the list of identified species.

Pomegranate (*Punicic granatum*) seed oil (PSO) is a rich source of FAs with conjugated double bonds. Cold-pressed PSO was transesterified, and the resulting mixture was analyzed by HPLC/MS. Many isomeric species with similar retention times tended to coelute. Still, the partial separation of the peaks allowed us to identify most of these lipids (Figure 7).

**Figure 8:**
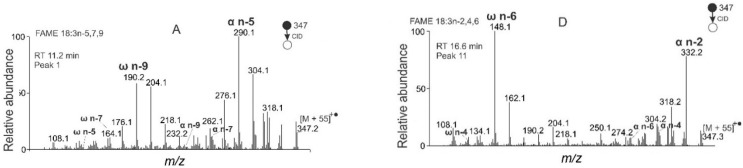

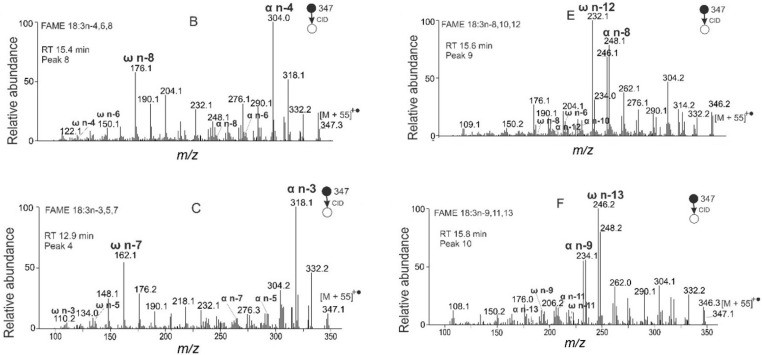
APCI MS/MS spectra of the [M + 55]^+^^•^ adducts of selected conjugated FAMEs from PSO interpreted as FAME 18:3n-5,7,9 (**A**), 18:3n-4,6,8 (**B**), 18:3n-3,5,7 (**C**), 18:3n-2,4,6 (**D**), 18:3n-8,10,12 (**E**), and 18:3n-9,11,13 (**F**).

All the abundant peaks corresponded to CLnAs. The highest intensity exhibited an isomer with t_R_ 11.2 min, which was interpreted as FAME 18:3n-5,7,9. Its MS/MS spectrum (Figure 8A) showed abundant diagnostic peaks *m/z* 190.2 and *m/z* 290.1, corresponding to an MBR value of 133. The spectrum closely matched the punicic acid methyl ester shown in Figure 2. Interestingly, two less abundant isomers with the same diagnostic fragments were detected at t_R_ 12.0 min and t_R_ 14.8 min (Supplementary Materials Figure S2). These species were isomers with the same double bond positions but different double bond geometries. The geometrical isomers of punicic acid, namely FAME 18:3n-5*t*,7*t*,9*c* (β-eleostearic acid); FAME 18:3n-5*c*,7*t*,9*t* (α-eleostearic acid), and FAME 18:3n-5*t*,7*t*,9*t* (catalpic acid) were detected in pomegranate seed oil previously [51,86,87,88,89]. As the elution of the FAs in reversed-phase systems proceeds from *cis* to *trans* isomers [20,90], the later eluting isomers likely contained a higher number of *trans* double bonds. The MS/MS spectra of FAMEs with three conjugated double bonds in different positions are shown in Figure 8B–F. In all of them, the MBR value was 133, and the diagnostic fragments allowed us to interpret them as FAME 18:3n-4,6,8 (Figure 8B), FAME 18:3n-3,5,7 (Figure 8C), FAME 18:3n-2,4,6 (Figure 8D), FAME 18:3n-8,10,12 (Figure 8E), and FAME 18:3n-9,11,13 (Figure 8F). The retention times of the latter two CLnAs were close to each other, which resulted in mixed spectra. Overall, ten CLnAs, one methylene-interrupted (18:2n-6,9), and two monounsaturated (18:1n-9 and 20:1n-9) FAMEs were identified in the PSO (Table 3). The results were in good agreement with previous analyses of PSO by silver-ion HPLC [51]. HPLC-based approaches to CLnAs analysis offer a higher number of isomers detected than GC [55,88,91,92]. We found four new CLnAs in the PSO, which, to the best of our knowledge, have not yet been described in the literature: two geometric isomers of 18:3n-2,4,6 (Figure 8D and Figure S2), FAME 18:3n-8,10,12 (Figure 8E), and FAME 18:3n-9,11,13 (Figure 8F). They are characterized by the double bonds closer to the terminal carbon end (FAME 18:3n-2,4,6) or methyl ester group (FAME 18:3n-8,10,12 and FAME 18:3n-9,11,13).

#### 2.3.3. FAMEs from *Marrubium vulgare* Seeds

**Figure 9:**
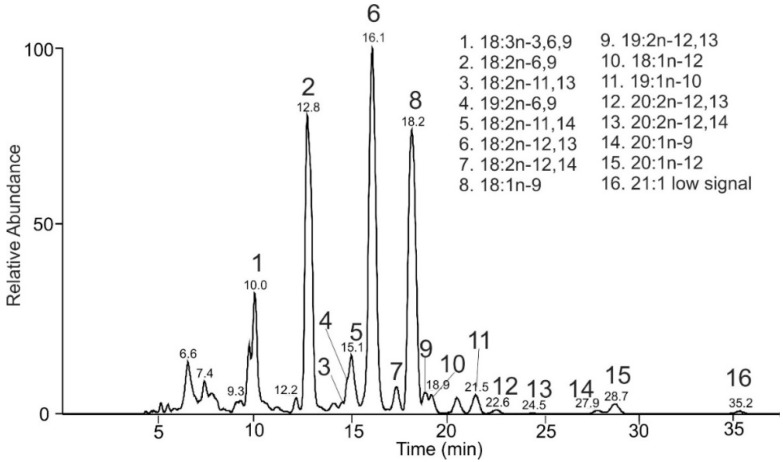
HPLC/APCI-MS base-peak chromatogram of FAMEs obtained from *Marrubium vulgare* seeds and the list of identified species.

White horehound (*Marrubium vulgare*) is a perennial, aromatic herb native to Europe, northern Africa, and southwestern and central Asia. Like other plants of the Lamiaceae family, it contains FAs with cumulated double bonds (allenic FAs). TGs from white horehound seeds were transesterified, and the resulting mixture of FAMEs analyzed by HPLC/MS (Figure 9). FAMEs with 18 to 21 carbons and up to three double bonds were detected.

**Figure 10:**
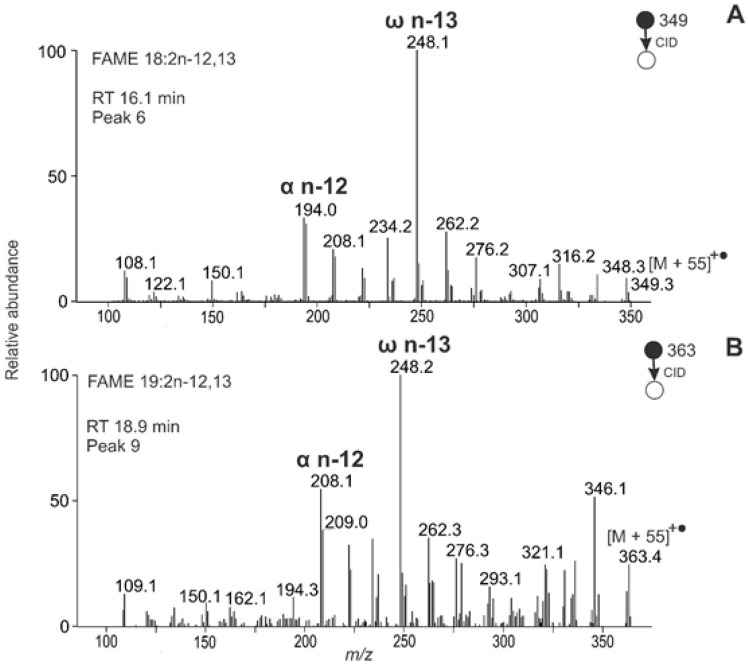
APCI MS/MS spectra of the [M + 55]^+^^•^ adducts of allenic FAMEs from *Marrubium vulgare* seeds interpreted as FAME 18:2n-12,13 (**A**) and 19:2n-12,13 (**B**).

The most abundant peak t_R_ 16.1 min corresponded to FAME 18:2 with the main fragments *m/z* 194.0 (α n-12) and *m/z* 248.1 (ω n-13), Figure 10A. The MBR value of 93 indicated two cumulated double bonds. It was interpreted as FAME 18:2n-12,13, most probably laballenic acid, highly abundant in *M. vulgare* seeds [61]. The fragmentation spectrum of FAME 18:2n-12,13 with the allenic system differed conspicuously from other arrangements of double bonds. The α fragment was accompanied by an α + 1 fragment with almost the same intensity, providing a double peak *m/z* 194/195 (Figure 10A). Analogous fragmentation behavior was also observed for other FAMEs with cumulated double bonds and helped us interpret allenic motifs in FAMEs. For instance, the compound eluting in 18.9 min was interpreted as FAME 19:2n-12,13. Its MS/MS spectrum provided *m/z* 208.1 (α n-12), *m/z* 209.0 (α n-12 + 1), and *m/z* 248.2 (ω n-13), corresponding to an MBR of 93 (Figure 10B). Analogously, peak t_R_ 22.6 min showing *m/z* 222.1 (α n-12), *m/z* 223.1 (α n-12 + 1), and *m/z* 248.2 (ω n-13) was consistent with 20:2n-12,13 (spectrum not shown).

**Figure 11:**
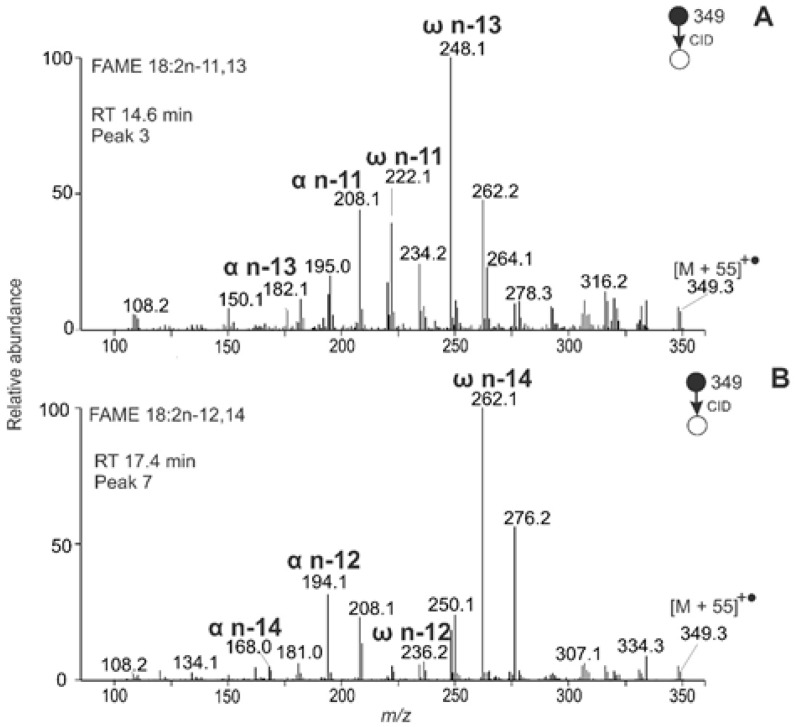
APCI MS/MS spectra of the [M + 55]^+^^•^ adducts of conjugated FAMEs from *Marrubium vulgare* seeds interpreted as FAME 18:2n-11,13 (**A**) and 18:2n-12,14 (**B**).

In addition to allenic species, *M. vulgare* seeds contained FAMEs with conjugated double bonds. For example, the chromatographic peak t_R_ 14.6 min represented FAME 18:2n-11,13. Its structure was deduced using *m/z* 182.1 (α n-13), *m/z* 208.1 (α n-11), *m/z* 222.1 (ω n-11), and *m/z* 248.1 (ω n-13), an MBR value of 107 (Figure 11A). Similarly, peak t_R_ 17.4 min corresponded to FAME 18:2n-12,14 (Figure 11B). Overall, sixteen unsaturated FAMEs were detected in *M. vulgare* seeds, including monounsaturated, diunsaturated with allenic and conjugated double bonds, and triunsaturated species with methylene-interrupted double bonds (Table 4).

#### 2.3.4. FAMEs from *Santalum album* Seeds

**Figure 12:**
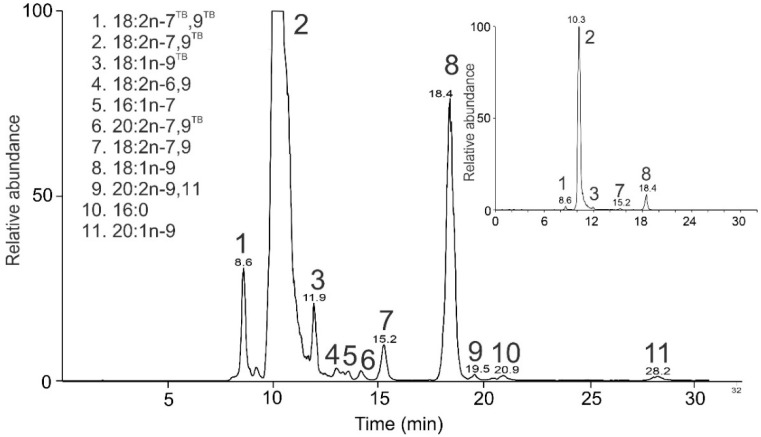
HPLC/APCI-MS base-peak chromatogram of FAMEs obtained from *Santalum album* seeds and the list of identified species. The inset shows a chromatogram of the same sample injected in 10× less amount.

Indian sandalwood (*Santalum album*) is a tropical tree native to southern India and Southeast Asia. The oil from its seeds and seeds of other Santalaceae species is a rich source of acetylenic FAs. [96]. FAMEs obtained by the transesterification of the TGs from *Santalum album* seeds provided chromatogram shown in Figure 12.

**Figure 13:**
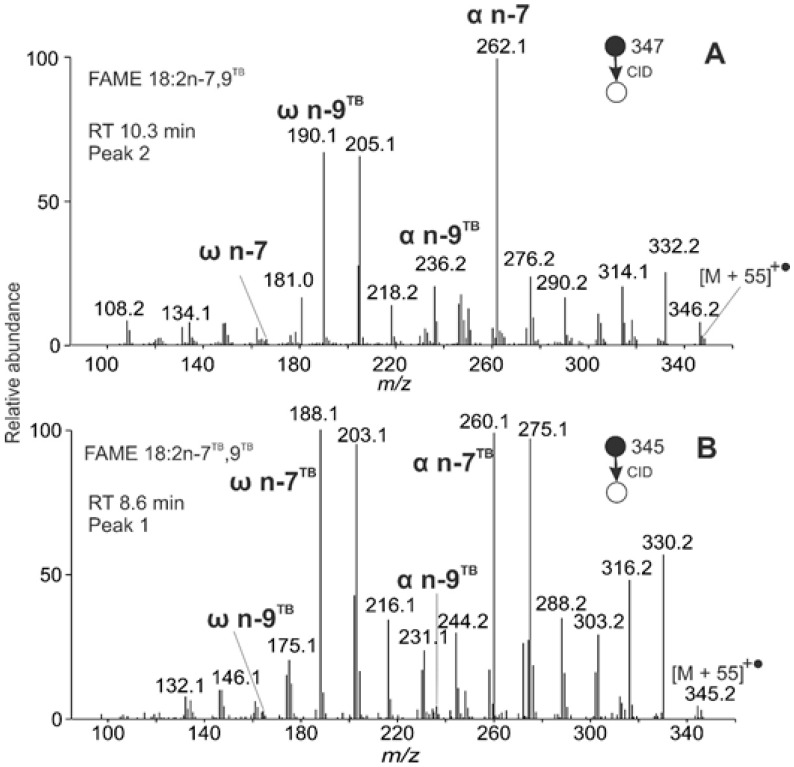
APCI MS/MS spectra of the [M + 55]^+^^•^ adducts of acetylenic FAME from *Santalum album* seeds interpreted as FAME 18:2n-7,9^TB^ (**A**) and 18:2n-7 ^TB^,9^TB^ (**B**).

The most abundant peak t_R_ 10.3 min corresponded to FAME with 18 carbons and either three double bonds or a double and a triple bond. The MS/MS spectrum (Figure 13A) revealed the latter possibility, i.e., an acetylenic acid methyl ester. Diagnostic fragment *m/z* 190.1 and its satellite ion *m/z* 205.1 indicated a triple bond in the n-9 position (ω n-9^TB^). The corresponding α fragment (α n-9^TB^) at *m/z* 236.1 was not accompanied by a significant satellite ion at *m/z* 251.1, likely because of the triple bond conjugation with the n-7 double bond. The α fragment *m/z* 262.0 and its satellite *m/z* 276.1 indicated a double bond in the position n-7. Low-intensity fragment ω n-7 was detected at *m/z* 166.1. The MBR value of 105 corresponds to a conjugated system of one double and one triple bond. The compound was identified as FAME 18:2n-7,9^TB^, most probably santalbic acid methyl ester.

The MS/MS spectrum of a peak in 8.6 min revealed another acetylenic FAME with two triple bonds (Figure 13B). The ω fragment *m/z* 188.1 and its satellite peak *m/z* 203.1 indicated the triple bond at the position n-9^TB^, and the α fragment *m/z* 260.1 and its satellite *m/z* 275.0 the triple bond in n-7^TB^. The complementary α (n-9^TB^) and ω (n-7^TB^) fragments *m/z* 236.1 and *m/z* 164.1, respectively, were of low abundance. The MBR value calculated from the most abundant fragments (*m/z* 188.1 and *m/z* 260.1) equaled 103 and was consistent with two conjugated triple bonds. The compound was identified as FAME 18:2n-7^TB^,9^TB^.

The seeds oil was also found to contain acetylenic acids (FAMEs 18:3n-9^TB^, 20:2n-7,9^TB^) and conjugated acids (FAMEs 18:2n-7,9, 20:2n-9,11) not reported previously for *S. album*. In summary, FAMEs with triple bonds conjugated with either double or triple bond were found, together with saturated, monounsaturated, diunsaturated conjugated and methylene interrupted, triunsaturated, and tetraunsaturated species (Table 5).

## 3. Experimental

### 3.1. Chemicals and Materials

MS-grade acetonitrile and methanol (Sigma-Aldrich, St. Louis, MO, USA) were used as received. Chloroform, hexane, and diethyl ether were distilled from analytical-grade solvents (Penta, Czech Republic). Other chemicals, NaCl (≥99%, Sigma-Aldrich, St. Louis, MO, USA), di-tert-butyl-4-methylphenol (Fluka, Buchs, Switzerland), Rhodamine 6G (Sigma-Aldrich, St. Louis, MO, USA), and Diazald (99%, Sigma-Aldrich, St. Louis, MO, USA) were used. The standards of crepenynic acid (99%) and punicic acid methyl ester (purity 98%) were from Larodan (Malmö, Sweden), and 9-octadecynoic acid methyl ester, 9(*E*),11(*E*)-octadecadienoic acid methyl ester, and 9(*Z*),11(*Z*)-octadecadienoic acid methyl ester (all 98%) were purchased from Cayman Europe (Tallinn, Estonia). The standards were dissolved in chloroform at 1 mg/mL concentrations and stored at −25 °C. *Bombus pratorum* males were collected in the Czech Republic during the spring season and immobilization at −18 °C. Cold-pressed pomegranate seed oil (organic, unrefined) was from Biopurus Ltd. (Ashford, England). Seeds of *Marrubium vulgare* and *Santalum album* were purchased from a local garden center.

### 3.2. Extraction and Transesterification of Lipids

The samples were treated with organic solvents to obtain total lipid extracts. Briefly, peripheral fat bodies of three *B. pratorum* males were dissected and extracted with CHCl_3_/CH_3_OH (1:1, *v*/*v*) containing di-tert-butyl-4-methylphenol at a concentration of 25 mg/mL (500 µL each) and sonicated for 15 min. The extract was collected using a Pasteur pipette. *M. vulgare* seeds (approx. 240 pieces; 0.25 g) or *S. album* seeds (5 pieces; 0.94 g) were crushed and extracted in methanol/chloroform (2:1 *v*/*v*, 10 mL) for 30 min. After filtration, 5 mL of 0.9% NaCl was added, shaken for few seconds, and the aqueous (upper) phase was removed. The cleaning step was repeated three more times with 2 mL of 0.9% NaCl solution.

Total lipid extracts or seed oil were separated by semipreparative TLC to isolate TGs.

Pre-cleaned, in-house made silica-gel glass TLC plates (60 mm × 76 mm) and hexane/diethyl ether (80:20, by vol.) mobile phase were used. TLC zones were made visible by spraying Rhodamine 6G solution (0.05% in ethanol). A zone corresponding to TGs (*B. pratorum* R_f_ = 0.36–0.55, pomegranate R_f_ = 0.20–0.55, *M. vulgare* R_f_ = 0.33–0.55, *S. album* R_f_ = 0.30–0.55) was scraped off the plate and extracted with 10 mL freshly distilled diethyl ether. The solvent was evaporated to dryness under a nitrogen stream.

While TGs from *B. pratorum*, pomegranate seed oil, and *M. vulgare* seeds were transesterified in acidic conditions [98], base-catalyzed transesterification [99] was required for *S. album* lipids containing triple bonds. FA standards were methylated by diazomethane (synthesized in-house from Diazald). Diazomethane in diethyl ether was added dropwise to the FA solution in chloroform (10 mg/mL) until the color of the reaction mixture turned light-yellow. Unreacted diazomethane was deactivated by formic acid.

### 3.3. RP-HPLC/APCI-MS and APCI-MS

The liquid chromatograph consisted of a Rheos Allegro UHPLC pump, Accela autosampler with an integrated column oven, and an LCQ Fleet ion-trap mass spectrometer; the system was controlled by Xcalibur software (all Thermo Fisher Scientific, San Jose, CA, USA). Develosil RP-Aqueous C30 (250 × 4.6 mm, particle size: 5 μm; Nomura Chemical, Seto, Japan) stainless-steel column and isocratic elution with acetonitrile at 0.7 mL/min flow rate [20] were used. The chromatography proceeded at laboratory temperature except for *B. pratorum* sample separated at 40 °C. The injected volume of samples (standards and biological samples, 1 mg/mL and 10–20 mg/mL, respectively) was 10–20 µL. The APCI vaporizer and heated capillary temperatures were set to 380 °C and 180 °C, respectively; the corona discharge current was 2 µA. Nitrogen served both as the sheath and auxiliary gas at a flow rate of 50 and 20 arbitrary units, respectively. The MS spectra of positively charged ions were recorded in the *m/z* 180–470 range. The CID MS^2^ spectra of [M + 55]^+•^ were collected using a data-dependent analysis with an isolation width of 1.7 Da and normalized collision energy of 28%. The *m/z* range of MS^2^ spectra was set automatically, depending on the precursor ion mass. The masses of the acetonitrile adducts for fragmentation were calculated as higher partners of the base peaks (*m/z* [M + H]^+^ + 54 Da). The retention times and relative peak areas were obtained from ion chromatograms extracted for [M + H]^+^. The high-resolution MS data were recorded using an LTQ Orbitrap XL hybrid mass spectrometer (Thermo Fisher Scientific, San Jose, CA, USA) equipped with an APCI ion source operated at the same conditions as for low resolution. The Orbitrap spectra were acquired at a resolution of 100,000 FWHM.

The standard (1 mg/mL) solutions were also analyzed by direct infusion to the mobile phase flow using the same APCI-MS conditions, as described above.

### 3.4. Fragment Ion Abbreviations and Nomenclature

The diagnostic ions in the MS/MS spectra of [M + 55]^+•^ were denoted “α” if they carried the ester moiety or “ω” if they contained the terminal-carbon end without the ester group. The double bond position was indicated as α n-x and ω n-x, where x is the distance from the terminal end of the hydrocarbon chain. A triple bond was marked by “TB” in superscript.

## 4. Conclusions

This work demonstrates the applicability of acetonitrile gas-phase chemistry in APCI for characterizing the structure of polyunsaturated FAMEs. The reaction of C_3_H_5_N^+•^ with double and triple bonds occurs in the ion source, and the reaction products are fragmented to generate diagnostic ions. The method is highly versatile and suitable to many (if not all) arrangements of double and triple bonds in mono- and polyunsaturated chains. It was successfully applied to FAMEs with isolated, cumulated, and conjugated double bonds, triple bonds, and their combinations. The localization of the isolated double and triple bond positions is straightforward because of intense α and ω fragments. Distinguishing a double bond from a triple bond is easy based on the satellite fragments. While the satellite ions appear at +14 Da in the lipids with a double bond, they are found as intense +15 Da fragments in the case of a triple bond. When two or more unsaturated bonds exist in a chain, the spectra predominantly show α and ω fragments related to cleavages of C–C bonds before and after the unsaturated region. This can be utilized for deducing a possible arrangement of unsaturated bonds. A parameter named multiple bond region (MBR) can be calculated using the most abundant fragments and compared to tabulated theoretical values. The type and position of the unsaturated bonds within the unsaturated region can then be inspected in detail after focusing on less intense diagnostic fragments and their satellites. In the case of allenic FAMEs, the α fragment was accompanied by an intense α + 1 fragment, which gave a hint for the cumulated double bonds. When a triple bond was present in a polyunsaturated chain, it manifested itself by the +15 Da satellite peak accompanying the corresponding diagnostic fragment.

The localization of unsaturated bonds by HPLC/APCI-MS/MS with an acetonitrile mobile phase is a simple and convenient method. Since the derivatization occurs in the ion source during ionization, there is no need to perform the chemical modification of the analytes as a separate step before the analysis. Nominal mass resolution spectra were successfully used for the structure elucidation. However, high-resolution MS/MS data could help distinguish α and ω fragments, thus making the interpretation even easier. In this work, unsaturated FAMEs were characterized in *Bombus pratorum*, *Punicum granatum*, *Marrubium vulgare,* and *Santalum album*. The method’s power is illustrated by the fact that, in addition to the known lipids, several new FAMEs were discovered. Although the method can also be applied to complex lipids [19,21,22], spectra interpretation is easier for lipids having only one fatty acyl chain.

## Figures and Tables

**Table 1:** Multiple bond region (MBR) values for common arrangements of double bonds (DBs) and triple bonds (TBs) in polyunsaturated chains.

MBR	Arrangement of Multiple Bonds
79	One triple bond –C≡C–
81	One double bond –CH=CH–
93	Two cumulated double bonds –CH=C=CH–
103	Two conjugated triple bonds –C≡C–C≡C–
105	One double bond and one triple bond, conjugated –CH=CH–C≡C–
107	Two conjugated double bonds –CH=CH–CH=CH–
119	One double bond and one triple bond, methylene-interrupted –CH=CH–CH_2_–C≡CH–
121	Two methylene-interrupted double bonds –CH=CH–CH_2_–CH=CH–
133	Three conjugated double bonds –CH=CH–CH=CH–CH=CH–
161	Three methylene-interrupted double bonds –CH=CH–CH_2_–CH=CH–CH_2_–CH=CH–
14n + 107	Two double bonds interrupted by several methylenes (–CH_2_–)_n_

**Table 2:** FAMEs identified in TG fraction of *B. pratorum* fat body lipids.

FAME	t_R_ (min)	Rel. Peak Area (%)	Literature Data (%) *
12:0	6.2	1.3	3.7 ± 4.2
14:1n-5	6.5	˂0.1	0.2 ± 0.3
18:3n-3,6,9	6.8	4.3	2.7 ± 0.3
16:1n-7	8.3	13.7	7.7 ± 1.9
16:1n-5	-	-	0.8 ± 0.4
14:0	8.4	1.7	5.4 ± 2.1
18:2n-6,9	8.5	0.7	0.8 ± 0.6
18:2n-3,6	8.5	0.3	-
17:1n-7	9.4	0.4	-
15:0	9.7	0.2	˂0.1
18:1n-7	10.5	0.6	-
18:1n-8	10.6	˂0.1	-
18:1n-9	10.6	0.3	-
18:1n-7	11.5	35.2	17.3 ± 3.6
18:1n-9	11.5	19.3	35.1 ± 3.2
16:0	12.0	5.5	18.5 ± 2.4
19:1n-9	13.5	2.6	-
19:1n-7	13.5	0.6	-
17:0	14.0	0.5	-
20:1n-7	16.9	0.8	˂0.1
20:1n-11	16.9	0.6	˂0.1
18:0	17.9	0.1	0.7 ± 0.3
19:0	21.4	˂0.1	-
24:2n-7,17	24.7	1.4	-
22:1n-7	25.2	0.3	-
20:0	28.0	˂0.1	0.2 ± 0.1
22:0	-	-	0.3 ± 0.1
25:2n-7,17	30.2	0.5	-
26:2n7,17	37.0	9.2	5.0 ± 2.4
23:0	38.5	˂0.1	-
24:1n-15	39.3	˂0.1	˂0.1
24:0	-	-	˂0.1
26:1n-17	-	-	0.2 ± 0.1

* Mean ± SD values of relative peak area values obtained by integrating GC/MS peaks; data for five bumblebee individuals. From ref. [41].

**Table 3:** FAMEs identified in TG fraction of pomegranate seed oil.

FAME	t_R_ (min)	Rel. Peak Area (%)	References
18:3n-5,7,9	11.2	36.0	[51,86,87,89,93,94,95]
18:3n-5,7,9	12.0	24.2	[51,86,87,89]
18:3n-4,6,8	12.3	2.5	[51]
18:3n-3,5,7	12.9	1.7	[51]
18:2n-6,9	13.6	1.5	[86,87,89,93,94,95]
18:3n-2,4,6	13.7	1.0	-
18:3n-5,7,9	14.8	9.4	[51,86,87,89]
18:3n-4,6,8	15.4	10.1	[51]
18:3n-8,10,12	15.6	3.3	-
18:3n-9,11,13	15.8	2.7	-
18:3n-2,4,6	16.6	2.3	-
18:1n-9	19.5	4.5	[86,87,89,93,94,95]
20:1n-9	30.1	0.8	[86,89]
14:0	-	-	[87,93]
16:0	-	-	[86,87,89,93,94,95]
18:0	-	-	[86,87,89,93,94,95]
18:3n-3,6,9	-	-	[95]
20:0	-	-	[86,87,89,95]
22:0	-	-	[93]
24:0	-	-	[86,87]
24:1	-	-	[86]

**Table 4:** FAMEs identified in TG fraction of *Marrubium vulgare* seed lipids.

FAME	t_R_ (min)	Rel. Peak Area (%)	References
18:3n-3,6,9	10.1	6.6	-
18:2n-6,9	12.8	25.4	-
18:2n-11,13	14.6	0.3	-
19:2n-6,9	14.9	1.2	-
18:2n-11,14	15.1	2.7	-
18:2n-12,13	16.1	31.4	[61]
18:2n-12,14	17.4	1.6	-
18:1n-9	18.2	26.5	-
19:2n-12,13	18.9	0.7	-
18:1n-12	19.2	0.6	-
19:1n-10	21.5	1.5	-
20:2n-12,13 *	22.6	0.4	[61]
20:2n-12,14 *	25.1	0.1	-
20:1n-9	27.9	0.2	-
20:1n-12	28.8	0.9	-
21:1 *	35.2	˂0.1	-
20:1n-11	-	-	[61]

* Tentative identifications.

**Table 5:** FAMEs identified in TG fraction of *S. album* seed lipids.

FAME	t_R_ (min)	Rel. Peak Area (%)	Literature Data (%) *
18:2 n-7^TB^,9^TB^	8.6	1.6	-
18:2n-7,9^TB^	10.3	89.0	33.5
18:1n-9^TB^	11.9	0.7	-
18:2n-6,9	13.0	0.2	1.5
16:1n-7	13.6	0.2	0.8
20:2n-7,9^TB^	14.2	0.2	-
18:2n-7,9	15.2	0.7	-
18:1n-9	18.4	7.0	52.1
20:2n-9,11	19.5	0.1	-
16:0	20.9	0.1	3.7
20:1n-9	28.2	0.2	-
16:1n-9	-	-	0.1
17:1	-	-	0.3
17:2	-	-	0.5
18:0	-	-	1.7
18:1n-7	-	-	1.4
18:3n-3,6,9	-	-	3.1
18:4n-3,6,9,12	-	-	1.3

* Composition of sandalwood oil ethyl esters reported in ref. [97].

## Data Availability

The data presented in this study are available on request from the corresponding author.

## Supplementary Materials

The following are available online, Figure S1: APCI MS/MS CID spectrum of the [M + 55]^+•^ adduct of ricinenic acid methyl ester (FAME 18:2n-7*c*,9*c*); MBR = 107; Table S1: High-resolution data for fragments from APCI MS/MS spectra of FAME standards. Figure S2: APCI MS/MS spectra of the [M + 55]^+•^ adducts of selected conjugated FAMEs from PSO interpreted as FAME 18:3n-5,7,9 (A), 18:3n-4,6,8 (B), 18:3n-2,4,6 (C), 18:3n-5,7,9 (D).

## Sample Availability

Samples of the compounds are not available from the authors.
