# Supplementary material for: Structural Characterization of Unusual Fatty Acid Methyl Esters with Double and Triple Bonds Using HPLC/APCI-MS2 with Acetonitrile In-Source Derivatization
Source: Molecules. 2021 Oct 26;26(21):6468. doi: 10.3390/molecules26216468 (PMC8588306; doi:10.3390/molecules26216468)
Supplement: Supplementary file 1 [file molecules-26-06468-s001.zip › molecules-1409725-supplementary.pdf]

Supplementary Materials

Structural Characterization of Unusual Fatty Acid Methyl Esters with Double and Triple Bonds Using HPLC/APCI-MS^2 with Acetonitrile In-Source Derivatization

Petra Horká [1,2], Vladimír Vrkoslav [1], Jiří Kindl [1], Karolina Schwarzová-Pecková [2] and Josef Cvačka [1,2]*

[1] Institute of Organic Chemistry and Biochemistry of the Czech Academy of Sciences, Flemingovo náměstí 542/2, 166 00 Praha 6, Czech Republic
[2] Charles University, Faculty of Science, Department of Analytical Chemistry, Hlavova 2030/8, 128 43 Prague 2, Czech Republic
* Correspondence: josef.cvacka@uochb.cas.cz; Tel.: +420 220 183 303

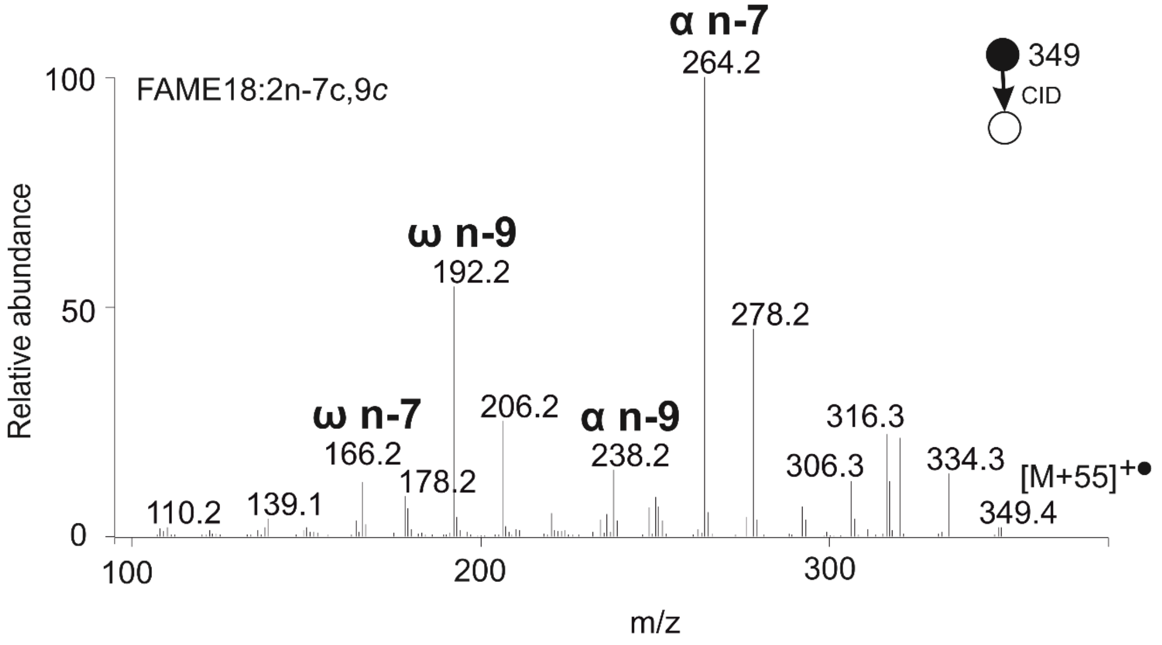

Figure S1. APCI MS/MS CID spectrum of the [M + 55]$^{+\bullet}$ adduct of ricinenic acid methyl ester (FAME 18:2n-7*c*,9*c*); MBR = 264 + 192 – 349 = 107.

Table S1. High-resolution data for fragments from APCI MS/MS spectra of FAME standards.

FAME	Fragment	Structure	Exact mass	Accurate mass	Delta ppm
FAME 18:2n-7*t*,9*t*	α n-7	$C_{16}H_{26}NO_2^{+\bullet}$	264.1958	264.1961	1.2
	α n-9	$C_{14}H_{24}NO_2^{+\bullet}$	238.1802	238.1803	0.6
	ω n-7	$C_{11}H_{20}N^{+\bullet}$	166.1590	166.1591	0.3
	ω n-9	$C_{13}H_{22}N^{+\bullet}$	192.1747	192.1748	0.7
FAME 18:3n-5*c*,7*t*,9*c*	α n-5	$C_{18}H_{28}NO_2^{+\bullet}$	290.2115	290.2113	-0.5
	ω n-9	$C_{13}H_{20}N^{+\bullet}$	190.1591	190.1590	-0.5
	α n-9 *	$C_{14}H_{24}NO_2^{+\bullet}$	238.1802	-	-
	ω n-5 *	$C_9H_{16}N^{+\bullet}$	138.1277	-	-
	α n-7 *	$C_{16}H_{26}NO_2^{+\bullet}$	264.1958	-	-
	ω n-7 *	$C_{11}H_{18}N^{+\bullet}$	164.1434	-	-
FAME 18:1n-9^{TB}	α n-9^{TB}	$C_{14}H_{22}NO_2^{+\bullet}$	236.1645	236.1647	0.7
	ω n-9^{TB}	$C_{13}H_{22}N^{+\bullet}$	192.1746	192.1748	0.7
	α n-9^{TB}+15	$C_{15}H_{25}O_2N^{+\bullet}$	251.1880	251. 1882	1.0
	ω n-9^{TB}+15	$C_{14}H_{25}N^{+\bullet}$	207.1982	207.1983	0.8
FAME 18:2n-6^{TB},9*c*	ω n-6^{TB}	$C_{10}H_{16}N^{+\bullet}$	150.1277	150.1278	0.4
	α n-6^{TB}	$C_{17}H_{26}NO_2^{+\bullet}$	276.1958	276.1961	1.1
	ω n-6^{TB}+15	$C_{11}H_{19}N^{+\bullet}$	165.1512	165.1512	0.2
	α n-6^{TB}+15	$C_{18}H_{29}NO_2^{+\bullet}$	291.2192	291.2196	1.0
	ω n-9	$C_{13}H_{20}N^{+\bullet}$	190.1590	190.1591	0.5
	α n-9 *	$C_{14}H_{24}NO_2^{+\bullet}$	238.1802	-	-

* Low intensity signal in the Orbitrap

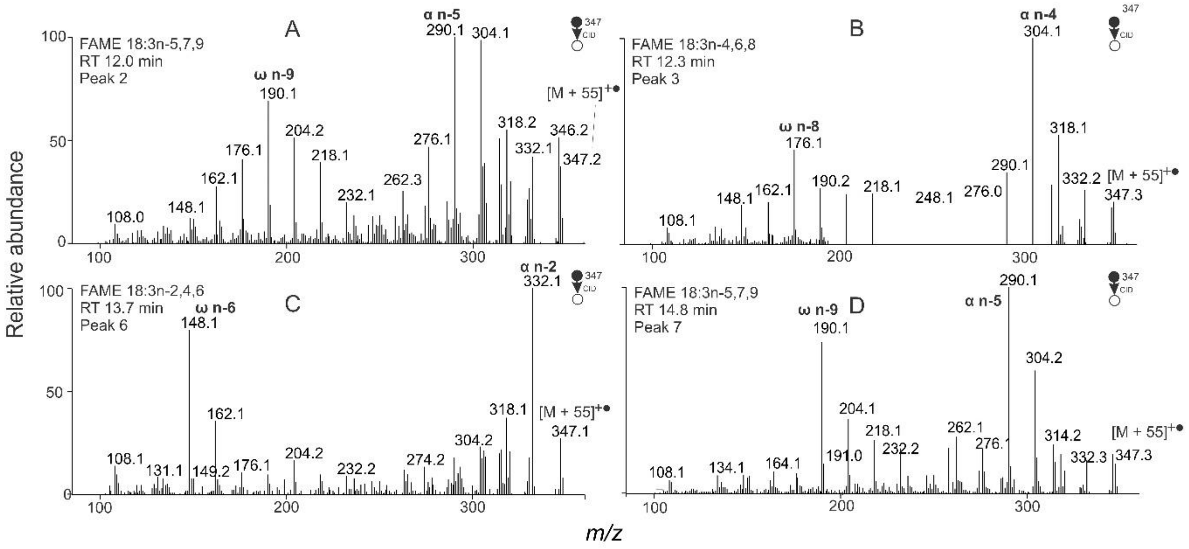

Figure S2: APCI MS/MS spectra of the [M + 55]$^{+\bullet}$ adducts of selected conjugated FAMEs from PSO interpreted as FAME 18:3n-5,7,9 (A), 18:3n-4,6,8 (B), 18:3n-2,4,6 (C), 18:3n-5,7,9 (D).
